# AI-Based Respiratory Monitoring-Guided Evaluation of Rottlerin Therapy for PRRS in Grower–Finisher Pig Farms

**DOI:** 10.3390/v18010072

**Published:** 2026-01-04

**Authors:** Cha Eun Yoon, Dong Hyun Cho, Hye Lim Park, Ju Yeon Song, Sangshin Park, Sang Won Lee, Yun Young Go, In-Soo Choi, Chang-Seon Song, Joong-Bok Lee, Seung-Yong Park, Yeong-Lim Kang

**Affiliations:** 1 Laboratory of Infectious Diseases, College of Veterinary Medicine, Konkuk University, Seoul 05029, Republic of Koreapaseyo@konkuk.ac.kr (S.-Y.P.); 2 Department of Applied Veterinary Medicine, College of Veterinary Medicine, Konkuk University, Seoul 05029, Republic of Korea; 3 QVet Co., Ltd., SKV1 Center, Seoul 02262, Republic of Korea; 4 Graduate School of Urban Public Health, University of Seoul, Seoul 02504, Republic of Korea

**Keywords:** porcine reproductive and respiratory syndrome virus (PRRSV), rottlerin, AI-based respiratory monitoring, SoundTalks^®^, grower–finisher pigs, antiviral therapy, oral fluid, field evaluation

## Abstract

Porcine reproductive and respiratory syndrome virus (PRRSV) remains a major cause of economic loss in the swine industry, and highly pathogenic variants such as NADC34-like PRRSV highlight the need for antiviral strategies that complement vaccination. In this field study, we evaluated the efficacy of AlimenWOW, a rottlerin–lipid formulation, in grower–finisher pigs under commercial conditions using AI-based respiratory monitoring. A total of 2000 pigs were assigned to four groups: AlimenWOW G1 (PRRSV-stable source farm), AlimenWOW G2 (PRRSV-unstable source farm), Control 1 (antibiotic), and Control 2 (antipyretic). Respiratory Health Status (ReHS) and a derived Clinical Cough Index (CCI = 100 − ReHS) were continuously recorded with SoundTalks^®^, and oral fluid PRRSV load, serology, clinical outcomes, and productivity were assessed over 4 weeks. AlimenWOW G2 showed a marked improvement in ReHS from severely compromised baseline values to levels comparable with healthy status, while both control groups remained low; CCI was significantly lower in AlimenWOW G2 than in controls from day 14 onward (*p* ≤ 0.0001). AlimenWOW treatment was associated with reduced PRRSV titers in oral fluid, lower mortality and wasting rates, and improved feed conversion with lower feed costs compared with controls. These findings indicate that AlimenWOW, integrated with AI-based acoustic monitoring, can improve respiratory health and mitigate PRRSV-associated clinical and economic losses, supporting its use as a complementary tool in PRRSV control programs.

## 1. Introduction

Porcine reproductive and respiratory syndrome virus (PRRSV) is an enveloped, positive-sense RNA virus of the family Arteriviridae that has become one of the most important pathogens impacting global swine production. Since its first description in North America and Europe in the late 1980s, PRRSV has spread worldwide and caused substantial economic losses in many pig-producing regions [1,2]. A recent analysis estimated an annual economic loss of approximately 1.2 billion USD in the United States alone [3,4]. Europe and Asia have similarly experienced major production losses, and a Korean farm-level study reported financial damage of roughly 30 billion KRW in PRRSV-affected herds [5,6,7]. Collectively, PRRSV is recognized as a major threat to the sustainability and profitability of the pig industry worldwide.

Clinically, PRRSV infection manifests differently in pregnant sows versus piglets and grower–finisher pigs. In pregnant sows, transplacental transmission leads to reproductive failure, with abortion, stillbirths, and mummified fetuses [8]. In piglets and growing pigs, PRRSV preferentially infects pulmonary alveolar macrophages, suppresses innate and adaptive immune responses, and induces interstitial pneumonia, growth retardation, and increased mortality [9,10]. The immunosuppressive nature of PRRSV infection also increases susceptibility to secondary bacterial and viral pathogens, thereby contributing to porcine respiratory disease complex (PRDC) [11,12]. Thus, PRRSV not only causes direct disease but also amplifies the impact of co-infections, exacerbating production losses.

In addition to viral and host factors, environmental conditions such as ambient temperature, humidity, and air quality influence PRRSV stability and transmission. Aerosolized PRRSV particles remain more stable at low temperatures, whereas their half-life decreases markedly as temperature rises [13]. Farms with a higher number of days with mean temperatures in the 4–10 °C range show an increased likelihood of PRRSV outbreaks, suggesting that cooler conditions favor viral persistence and airborne spread [14]. Under high ambient temperatures, anaerobic decomposition of manure in slurry pits beneath the pen floor increases the production of noxious gases such as ammonia (NH_3_) and hydrogen sulfide (H_2_S) [15,16]. These gases can damage the respiratory mucosa, impair mucociliary clearance, and trigger inflammation, thereby aggravating the clinical course of PRRSV infection [17,18,19,20]. PRRSV-infected pigs, which already have compromised pulmonary macrophage function, are particularly vulnerable to respiratory damage and secondary bacterial complications in such environments [21,22].

Among the emerging PRRSV variants, NADC34-like PRRSV has attracted particular attention. This virus belongs to PRRSV-2 lineage 1.5 and was first reported in 2014 at the National Animal Disease Center in Iowa, USA [23,24]. Since then, its spread has been documented in China and Korea [25,26]. In Korea, NADC34-like PRRSV was initially identified in July 2022 during an outbreak on a farm in Hongseong, Chungcheongnam-do, characterized by high sow mortality and abortion storms. Whole-genome sequencing performed at the Animal Disease Diagnostic Center of Jeonbuk National University revealed 90.1% nucleotide similarity to IA/2014/NADC34 and suggested putative recombination events in several genomic regions [26,27]. Korean NADC34-like isolates have a genome length of approximately 15,088 bp and appear to have originated from recombination between a commercial RespPRRS MLV vaccine strain and the NADC34 lineage. These isolates share a continuous 100-amino-acid deletion in NSP2, identical to that of IA/2014/NADC34, and this deletion has been proposed as a molecular marker of highly pathogenic PRRSV [26]. National surveillance data reported 12 abortion storms in 2022 and PRRSV infections across 34 farms (2124 pigs) in 2023, demonstrating the significant impact of NADC34-like PRRSV in Korea.

Currently, vaccination is the mainstay of PRRSV control. Commercial vaccines include killed virus (KV) and modified live virus (MLV) formulations [28]. KV vaccines offer superior safety but often provide limited immunogenicity and suboptimal protection [29]. MLV vaccines can afford robust protection against homologous or closely related strains, but cross-protective efficacy against diverse field strains is frequently incomplete [30,31,32]. Furthermore, MLV strains may persist in vaccinated animals, be shed into the environment, and undergo recombination, raising concerns about genetic variation and potential changes in virulence [33,34]. The emergence of highly pathogenic variants that partially escape vaccine-induced immunity has been reported [35], highlighting the need for additional antiviral strategies to complement vaccination [36].

Natural product–derived compounds have emerged as promising candidates for novel antiviral interventions. Rottlerin is a natural polyphenol isolated from the fruit of the kamala tree (*Mallotus philippensis*), traditionally used for its anti-inflammatory and antiparasitic properties, and more recently reported to possess anticancer and antioxidant activities [37,38]. Early studies suggest that rottlerin modulates cytoskeletal dynamics and endocytic pathways, including PKCδ-related signaling, and partially inhibits macropinocytosis-mediated entry of PRRSV [39,40,41]. Antiviral effects have also been documented against Zika virus and feline coronavirus [42,43], suggesting potential broad-spectrum activity. However, the low aqueous solubility of rottlerin limits its in vivo application [44]. Lipid nanoparticle (LNP) or lipid mixture–based formulations can improve solubility, enhance stability, and facilitate cellular delivery, thereby maximizing antiviral efficacy [43,45].

At the same time, advances in precision livestock farming (PLF) have enabled more objective and continuous monitoring of herd health. The AI-based acoustic system SoundTalks^®^ automatically detects and quantifies cough events in pig barns and converts them into a numerical respiratory health index, the Respiratory Health Status (ReHS) score [46,47]. This system uses deep learning–based convolutional neural networks (CNNs) to distinguish cough sounds from background noise under diverse farm conditions [48,49] and displays ReHS scores and alerts for farm managers [50,51,52,53]. A CNN–RNN–based cough detection system has been reported to achieve 99.6% accuracy and 99.7% specificity in real farm environments [54,55,56], indirectly supporting the reliability of ReHS as a proxy for respiratory health.

In this context, AlimenWOW, a rottlerin–lipid formulation, was developed as a practical veterinary product intended to deliver rottlerin efficiently to the lungs and to interfere with PRRSV entry into porcine alveolar macrophages. The present field study aimed to evaluate, under commercial grower–finisher conditions, the effect of AlimenWOW on respiratory health (ReHS and AI-derived clinical cough index), mortality and wasting, PRRSV viral load, antibody responses, and economic performance in herds with different PRRSV stability status.

## 2. Materials and Methods

### 2.1. Preparation of the Rottlerin-Lipid (AlimenWOW) Mixture

The rottlerin–lipid mixture was prepared using a modified thin-film hydration method based on [40]. Briefly, kamala powder and lecithin were mixed at a 1:11 (*w*/*w*) ratio and dispersed in ethyl acetate, followed by repeated filtration. The filtrate was subjected to bioassays, which confirmed an approximately 1.5-log reduction in PRRSV titer. The mixture was then recovered by thin-film formation, yielding a product containing 70 mg rottlerin per g of mixture. The dried product was subsequently blended with trehalose at a 1:4 (*w*/*w*) ratio, resulting in a final rottlerin concentration of 14 mg/g. This rottlerin–lipid mixture was used as the basis for the investigational product AlimenWOW. The final field formulation was manufactured using the same process by an industrial partner (QVet Co., Ltd., Seoul, Republic of Korea) and supplied for the trial.

### 2.2. Farm, Animals, and Study Design

The clinical trial was conducted in a commercial grower–finisher farm located in Jeolla Province, Korea, under AI-based smart-farm conditions. A total of 2000 approximately 12-week-old three-way crossbred pigs (Yorkshire × Landrace × Duroc) were enrolled. Pigs were introduced weekly in cohorts of 500 animals, each cohort defined as a batch, resulting in four batches.

**Figure 1:**
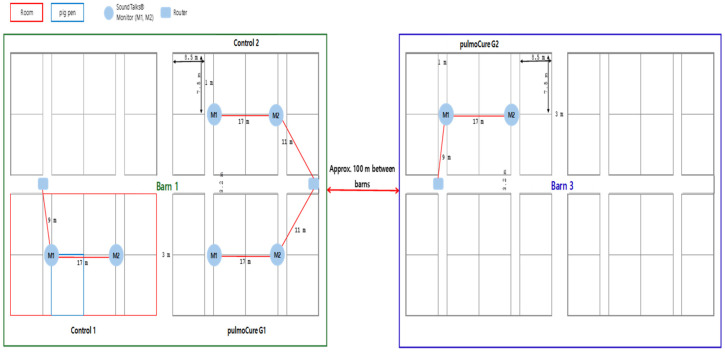
Schematic layout of pig pens and SoundTalks^®^ device positions for experimental groups and controls. AlimenWOW G1, Control 1 (antibiotic), and Control 2 (antipyretic) groups were housed within the same barn (Barn 1; green square) under the supervision of a single caretaker, whereas the AlimenWOW G2 group was raised in a separate barn (Barn 3; blue square) managed independently by another caretaker. The two barns were located approximately 100 m apart, and their air flow, entry routes, and personnel were kept completely independent to minimize the possibility of cross-contamination between groups. Each pig pen was equipped with SoundTalks^®^ AI-based respiratory monitoring devices (M1 and M2) and a router to continuously (24 h) record respiratory health status (ReHS) throughout the study period.

One treatment group (AlimenWOW G1, *n* = 500) consisted of pigs originating from a PRRSV-stable source farm. Three additional groups originated from a PRRSV-unstable source farm and were assigned as follows: AlimenWOW G2 (*n* = 500), Control 1 (antibiotic, *n* = 500), and Control 2 (antipyretic, *n* = 500). AlimenWOW G1, Control 1, and Control 2 were housed in the same barn (Barn 1) and managed by the same stockperson. AlimenWOW G2 was housed in a separate barn (Barn 3) and managed independently. The two barns were located approximately 100 m apart at their nearest point and were operated as separate units in terms of airflow, traffic flow, and personnel (Figure 1).

Immediately after placement and group allocation, pigs in the AlimenWOW groups received 10 mg/kg of AlimenWOW once daily via the drinking water for 1–3 consecutive days. An additional single dose was administered around day 7 in response to clinical deterioration signals detected by the SoundTalks^®^ monitoring system, as part of routine animal welfare–oriented farm management rather than for efficacy assessment.

Medicated water was delivered using a dosing pump adjusted so that the full dose would be consumed over approximately 4 h.

In Control 1, pigs received amoxicillin via drinking water under conditions comparable to those of the AlimenWOW groups. When red ReHS alerts occurred, ceftiofur was administered intramuscularly according to the same animal welfare–oriented farm management principles.

In Control 2, antipyretic agents were administered in drinking water at all treatment time points according to the farm’s standard management protocol. All animal procedures were approved by the Institutional Animal Care and Use Committee of Konkuk University (KUIACUC; approval no. KU25145).

### 2.3. AI-Based Respiratory Health Monitoring

An AI-based acoustic monitoring device (SoundTalks^®^, SoundTalks NV, Leuven, Belgium) was procured from FarmZen Co., Ltd. (Daegu, Republic of Korea) and installed in the barns to continuously monitor respiratory health. The SoundTalks^®^ system was used as a monitoring tool to support clinical management decisions by enabling early detection of respiratory deterioration, and was not used as a direct measure of treatment efficacy. SoundTalks^®^ automatically detects cough sounds, distinguishes them from background noise, and compares noise profiles between the current and previous day to calculate a Respiratory Health Status (ReHS) score ranging from 0 (worst) to 100 (best), which is displayed on a web-based dashboard. Under normal conditions, ReHS scores between 60 and 100 are displayed in green, indicating a healthy status. Yellow and red indicators denote potential or overt respiratory risk and correspond to ReHS ranges of 40–59 and 0–39, respectively. In emergency situations, a red warning light is activated on the in-barn monitor, and a corresponding alert appears on the website.

Because a single device can reliably monitor up to approximately 20 m, and the length of each room exceeded this distance, two SoundTalks^®^ units were installed per batch. Devices were mounted on walls without doors or ventilation fans to minimize airflow disturbances. To reduce installation-related effects, all devices were installed at least 48 h prior to pig placement. Data acquisition was maintained continuously throughout the trial. ReHS was calculated based on SoundTalks^®^ acoustic monitoring data and was used to quantitatively describe overall trends in respiratory health status at the batch level, using the mean value derived from two devices installed per batch to account for spatial coverage limitations of the monitoring system.

To quantify clinical cough severity, we derived an inverse index of respiratory impairment based on ReHS, defined as (100 − ReHS). The resulting Clinical Cough Index (CCI) is inversely correlated with ReHS and serves as a clinical indicator of respiratory symptom severity derived from SoundTalks^®^ acoustic monitoring data.

### 2.4. Sample Collection

Each experimental group was followed for approximately one month from the day of placement (day 1). Oral fluid (OF) and blood samples were collected three times per batch: on day 1 (baseline), and days 14 and 28 after initial treatment.

Oral fluid sampling was performed at the pen level to noninvasively monitor viral circulation. One cotton rope was suspended for every two pens (four ropes per batch) and left in place for 30 min to allow free chewing by the pigs. After adequate chewing, ropes were collected individually, and approximately 5 mL of OF was recovered from each.

In parallel, blood sampling was carried out to evaluate viremia and serological responses at the individual level. In each batch, eight pigs (one per pen) were randomly selected, and approximately 3 mL of whole blood was collected from the jugular vein. Blood samples were centrifuged at 3000× *g* rpm for 10 min to obtain serum.

Batch allocation was based on weekly cohorts of 500 pigs and their baseline respiratory status and PRRSV load. Batches with a day-1 ReHS ≥ 70 (classified as “stable”) and a baseline PRRSV load of approximately 0.1 log_10_ TCID_50_/mL were assigned to AlimenWOW G1. Three batches with day-1 ReHS ≤ 30 (classified as “risk”) and baseline viral loads of approximately 1.5 log_10_ TCID_50_/mL were selected and allocated to AlimenWOW G2, Control 1, and Control 2, respectively.

### 2.5. PRRSV Antigen Detection

Since OF represents pooled secretions from multiple animals and typically contains low viral concentrations, OF samples were concentrated prior to analysis to enhance sensitivity as described previously [57,58,59]. Viral nucleic acids were extracted from OF and serum using the genC™ vNA Extraction Kit (Neuclacid Inc., Seoul, Republic of Korea) according to the manufacturer’s protocol.

Extracted RNA was subjected to reverse transcription quantitative PCR (RT–qPCR) targeting the PRRSV ORF7 gene using the genP™ PRRSV RT–qPCR Kit (Neuclacid Inc., Seoul, Republic of Korea). Reactions were prepared according to the manufacturer’s protocol with 10 μL of PRRSV MM, 5 μL of PRRSV PM, and 5 μL of RNA template per reaction. RT–qPCR cycling conditions are summarized in Table 1. Real-time PCR was performed on a CFX96 Dx Real-Time PCR Detection System (Bio-Rad Laboratories, Inc., Hercules, CA, USA).

Samples with a threshold cycle (Ct) value ≤ 35 were considered PRRSV-positive according to the kit manual. Viral titers were calculated as TCID_50_/mL using the Reed–Muench method [40].

### 2.6. Serological Analysis

Serum samples collected for antigen testing were also used for individual serology. PRRSV-specific antibodies were measured using the PRRS XR ELISA—Porcine Reproductive and Respiratory Syndrome Ab test kit (BioCheck UK Ltd., Winkfield Row, UK). All procedures were performed according to the manufacturer’s instructions. Samples with ELISA S/P ratio ≥ 0.5 were classified as PRRSV antibody–positive.

### 2.7. Clinical Outcome Scoring

Clinical outcomes were assessed as described for the grower–finisher outcome analysis. Mortality was recorded daily and expressed as the percentage of pigs that died during the trial relative to the total number of pigs placed in each group.

Wasting rate was quantified using a three-tier clinical grading system that integrated respiratory signs, body condition, and activity. Pigs with severe coughing or abdominal (labored) breathing were categorized as grade A; pigs showing these signs plus prominent spinal processes and abdominal emaciation were categorized as grade B; and pigs with grade B signs plus marked reduction in activity and inability to stand (lateral recumbency) were categorized as grade C. Pigs with grade A or higher were defined as wasted, and wasting rates were calculated for each group.

To further quantify disease severity, a five-point composite clinical score system was used: 1 point for pigs with cough detectable in ReHS, 2 points for abdominal breathing, 3 points for protruding spine and abdominal wasting, 4 points for non-ambulatory pigs, and 5 points for dead pigs. The number of pigs in each category was recorded for each group and each week (Supplementary Table S1), and the distribution of scores was used to compare overall clinical severity.

### 2.8. Economic Analysis

Economic analysis was performed using productivity and cost-related variables collected from AlimenWOW G2, Control 1, and Control 2. AlimenWOW G1 was excluded from economic comparisons because pigs originated from a PRRSV-stable source farm and differed from the other groups in baseline infection risk and immune status.

The variables included initial and final total body weight (used to derive total weight gain), total feed intake during the rearing period, mean days on feed, mean slaughter age, average daily gain (ADG), feed conversion ratio (FCR), feed cost per kg of gain, contract fee per head, total cost per head, and finishing rate.

Total weight gain was calculated as the difference between initial and final total body weight and divided by mean days on feed to obtain ADG. FCR was calculated as total feed intake divided by total weight gain. Total feed cost was derived by multiplying total feed intake by unit feed price and converted to feed cost per kg of gain. Contract fee per head was determined according to the farm’s contract scheme, and total cost per head was calculated as the sum of feed cost and contract fee.

Slaughter age analysis was based on the individual slaughter age of each pig. Mean slaughter age was calculated, and pigs were categorized as slaughtered at or before 191 days versus after 191 days. The mean slaughter age of AlimenWOW G2 (191 days) was used as a reference. Feed costs during the 14-day interval from 177 to 191 days were calculated separately to compare feed expenditure between groups over the same period.

As an indicator of environmental stability, the number of days with ReHS ≥ 60 was used as an auxiliary variable. Although ReHS was not directly incorporated into productivity or cost indices, it was used to interpret the stability of the rearing environment.

### 2.9. Statistical Analysis

All statistical analyses and plots were generated using GraphPad Prism 10 (GraphPad Software, San Diego, CA, USA) and SAS 9.4 (SAS Institute Inc., Cary, NC, USA). One-way analysis of variance (one-way ANOVA) was used for group comparisons. Where ANOVA indicated significance, Tukey’s multiple comparison test was applied as a post hoc test. Data are presented as mean ± standard deviation (SD). Differences were considered statistically significant at *p* ≤ 0.05. Significance levels were denoted as follows: *p* < 0.05 (*), *p* < 0.01 (**), *p* < 0.001 (***), and *p* < 0.0001 (****).

## 3. Results

### 3.1. Effect of AlimenWOW on AI-Based Respiratory Health Indices

Based on baseline ReHS, batches were assigned to AlimenWOW G1 (ReHS ≥ 70; PRRSV-stable origin) or AlimenWOW G2 (ReHS ≤ 30; PRRSV-unstable origin), while groups receiving antibiotics or antipyretics according to the farm’s routine program were designated as Control 1 and Control 2, respectively.

**Figure 2:**
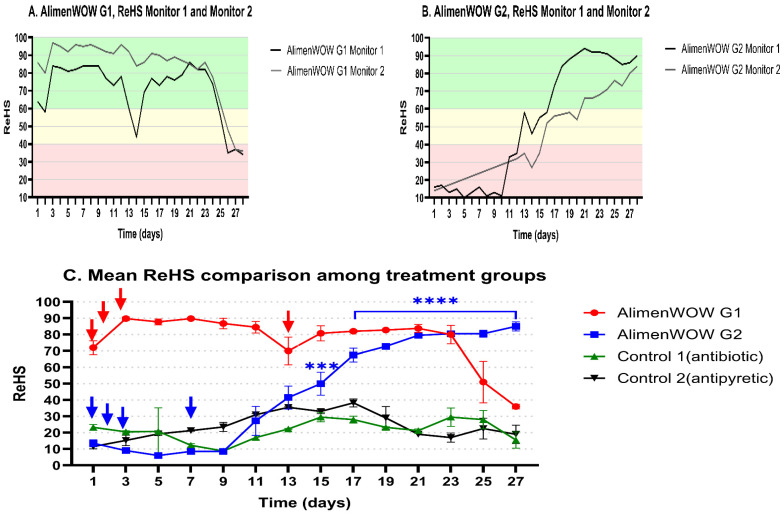
Respiratory health status (ReHS) monitored by an AI-based sound analysis system following AlimenWOW administration. Panels (**A**,**B**) show ReHS values obtained from two independent monitors for AlimenWOW G1 and G2, respectively. Panel (**C**) presents mean ReHS values for each group, confirming consistent trends across monitors. Red and blue arrows indicate the dosing schedules for AlimenWOW G1 and G2, respectively. Asterisks indicate statistical significance (*** *p* < 0.001, **** *p* < 0.0001).

Respiratory health was monitored over 4 weeks using SoundTalks^®^ (Figure 2). In AlimenWOW G1, ReHS values were ~80 immediately after treatment and remained high for approximately 22 days. A gradual decline was observed during the final 6 days of the trial, but ReHS remained within the “healthy” or mildly affected range. In AlimenWOW G2, ReHS values remained ≤30 during weeks 1–2 but increased to 60–80 during weeks 3–4. In contrast, Control 1 and Control 2 maintained low ReHS values of 20–30 throughout the observation period (*p* < 0.0001).

AlimenWOW G1 and the control groups were housed in Barn 1, whereas AlimenWOW G2 was housed in Barn 3. Nevertheless, ReHS trends were consistent within each barn, with AlimenWOW-treated groups showing higher ReHS than the control groups. In particular, AlimenWOW G2 exhibited a marked improvement in ReHS. These findings suggest that the administration of AlimenWOW had a greater impact on respiratory health than barn-level environmental differences.

Minor variation in body-weight distribution between pens was observed; however, no significant difference was detected between individual devices, and group-specific patterns were consistent. Therefore, ReHS values from the two devices per batch were averaged to obtain representative scores. Analysis of mean ReHS confirmed significant differences between AlimenWOW-treated groups and both control groups (Figure 2C, *p* < 0.001).

### 3.2. Effect of AlimenWOW on Mortality and Wasting

Mortality and wasting rates for all groups are summarized in Table 2. During week 1, no deaths occurred in AlimenWOW G1, whereas 1 death was recorded in AlimenWOW G2; 2 and 7 deaths occurred in Control 1 and Control 2, respectively. In week 2, 1, 2, 3, and 11 deaths occurred in AlimenWOW G1, AlimenWOW G2, Control 1, and Control 2, respectively. In week 3, no deaths occurred in AlimenWOW G1, while 2 deaths occurred in AlimenWOW G2 and 5 deaths each in Control 1 and Control 2. In week 4, 1 death occurred in each AlimenWOW group, 3 deaths in Control 1, and 10 deaths in Control 2.

Cumulatively, mortality rates were 0.4% in AlimenWOW G1 and 1.2% in AlimenWOW G2, compared with 2.6% in Control 1 and 6.6% in Control 2. Thus, mortality was at least 5.5-fold higher in Control 2 than in AlimenWOW-treated groups, and 2.1-fold higher in Control 1 than in AlimenWOW G2.

Wasting rates were also lower in the AlimenWOW groups. In week 1, 4 wasted pigs were recorded in AlimenWOW G1 and none in AlimenWOW G2, whereas 2 and 6 wasted pigs were observed in Control 1 and Control 2, respectively. No wasted pigs were recorded in either AlimenWOW group in week 2, while 2 wasted pigs were recorded in Control 1 and none in Control 2. In week 3, 2 wasted pigs were observed in AlimenWOW G1 and none in AlimenWOW G2, compared with 5 wasted pigs in Control 1 and none in Control 2. In week 4, no wasting was recorded in either AlimenWOW group, whereas 0 and 3 wasted pigs were observed in Control 1 and Control 2, respectively.

Overall wasting rates were 1.2% in AlimenWOW G1, 0% in AlimenWOW G2, and 1.8% in both Control 1 and Control 2.

### 3.3. Effect of AlimenWOW on Clinical Cough Dynamics

Cough-related clinical signs were quantified using the Clinical Cough Index (CCI), derived from the inverse of ReHS (100 − ReHS). In field trials, demonstrating vaccine or drug efficacy using traditional clinical cough scores is challenging [60,61]; therefore, we used the SoundTalks^®^-based CCI as an objective proxy for clinical respiratory severity.

**Figure 3:**
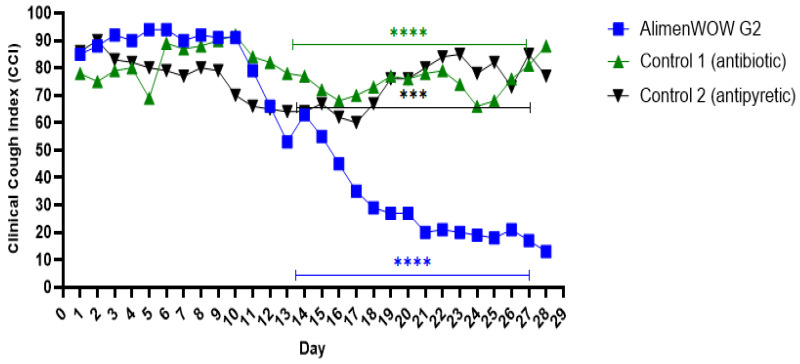
Clinical Cough Index (CCI) after investigational AlimenWOW administration. The ReHS (Respiratory Health Status) values obtained from the AI-based cough monitoring system (SoundTalks^®^) were inverted to the Clinical Cough Index (CCI = 100 − ReHS) to express the degree of clinical respiratory symptom manifestation. Asterisks indicate statistical significance (*** *p* < 0.001, **** *p* < 0.0001).

AlimenWOW was administered at 10 mg/kg via drinking water once daily from days 1–3, with an additional single dose on day 7 triggered by a SoundTalks^®^ alarm. Following the second administration, AlimenWOW G2 showed a clear improvement in ReHS and a corresponding reduction in CCI (Figure 3).

During the early phase of the trial (days 1–14), there were no significant differences in CCI among AlimenWOW G2, Control 1, and Control 2. However, from day 14 to day 28, CCI remained significantly lower in AlimenWOW G2 than in both control groups (*p* ≤ 0.0001). Notably, AlimenWOW G2 exhibited a rapid decline in CCI after the second administration on day 7, indicating a marked reduction in cough frequency and clinical respiratory manifestations.

AlimenWOW-treated group (G2) maintained significantly lower CCI values compared with the Control 1 (antibiotic) and Control 2 (antipyretic) groups after day 14 (*p* ≤ 0.001, *p* ≤ 0.0001), indicating that AlimenWOW alleviated clinical respiratory symptoms PRRSV infection.

Clinical score distributions mirrored the ReHS trends, with AlimenWOW-treated groups showing predominantly mild clinical signs compared with controls. Detailed score distributions across groups and time points are provided in Supplementary Table S1.

### 3.4. Reduction of PRRSV Load in Oral Fluid

To determine whether improved respiratory health following AlimenWOW administration was associated with reduced PRRSV burden, we quantified viral loads in OF samples. One AlimenWOW-treated group (AlimenWOW G1) originated from a PRRSV-stable farm, whereas three groups from a PRRSV-unstable farm were assigned as AlimenWOW G2, Control 1, and Control 2.

North American–type PRRSV titers were expressed as log_10_ TCID_50_/mL. In AlimenWOW G1, viral loads remained at 0–0.1 log_10_ TCID_50_/mL on days 1, 14, and 28, effectively negative. In AlimenWOW G2, viral titers were approximately 1.2–1.5 log_10_ TCID_50_/mL on day 1 and increased to 2.3–2.5 log_10_ TCID_50_/mL by day 14. At this time point, viral loads in AlimenWOW G2 were significantly lower than in Control 1 and Control 2 (each ~2.8–3.0 log_10_ TCID_50_/mL; *p* < 0.0001). By day 28, PRRSV was undetectable (0 log_10_ TCID_50_/mL) in all pigs in AlimenWOW G2.

**Figure 4:**
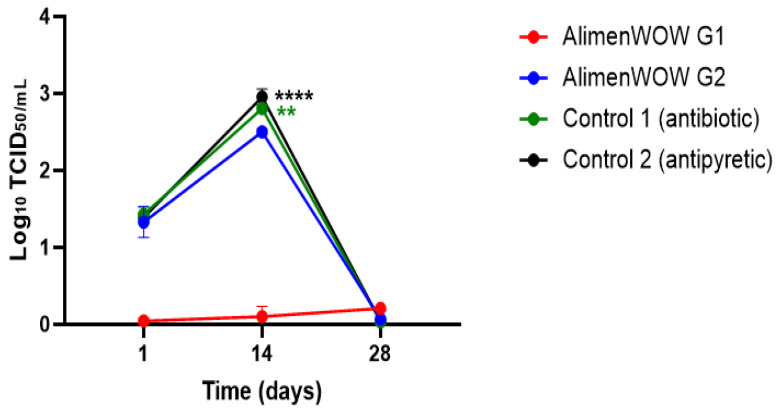
Viral titers (Log_10_ TCID_50_/mL) of PRRSV NA-type detected in oral fluid (OF) after investigational AlimenWOW administration. The AlimenWOW G1 group maintained near-undetectable viral titers throughout Day 1, Day 14, and Day 28. The AlimenWOW G2 group showed an increase in viral titers from Day 1 to Day 14; however, the titers at Day 14 were significantly lower than those of Control 1 (antibiotic; green line) and Control 2 (antipyretic; black line). Control 1 and Control 2 exhibited the highest viral titers at Day 14, and all groups showed very low or near-undetectable levels by Day 28. *p* < 0.01, *p* < 0.0001 (compared with each control group). Asterisks indicate statistical significance (** *p* < 0.01, **** *p* < 0.0001).

In Control 1 and Control 2, viral loads were approximately 1.3–1. log_10_ TCID_50_/mL on day 1, increased to ~2.8–3.0 log_10_ TCID_50_/mL on day 14, and decreased to 0 log_10_ TCID_50_/mL by day 28 (Figure 4). All groups tested negative for European-type PRRSV throughout the study.

### 3.5. Effect of AlimenWOW on PRRSV-Specific Antibody Responses

**Figure 5:**
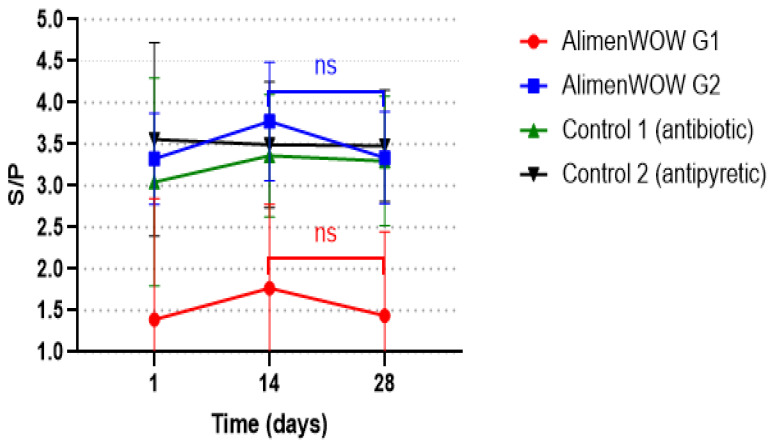
PRRSV-specific antibody responses measured by ELISA (S/P ratio) in AlimenWOW-treated and control groups at days 1, 14, and 28 after treatment. Data are presented as mean with error bars. Statistical significance is indicated in the figure (ns, not significant).

To indirectly assess the relationship between viral reduction and immune response, we examined longitudinal changes in PRRSV-specific antibody levels. In AlimenWOW G2, the mean ELISA S/P ratio was approximately 3.3 on day 1, increased to ~3.7 on day 14 (peak response), and declined by day 28 to levels similar to baseline (~3.3) (Figure 5).

AlimenWOW G1 showed a broadly similar kinetic pattern, though at lower absolute S/P values, reflecting its origin from a PRRSV-stable farm. Control 1 showed S/P values of ~3.0 on day 1, a slight increase by day 14, and little change by day 28. Control 2 maintained relatively constant S/P values between 3.4 and 3.6 throughout the trial.

Among all groups, AlimenWOW G2 exhibited the most pronounced temporal change in antibody levels, with a clear rise by day 14 followed by a decline by day 28, consistent with an active response to decreasing antigen burden.

### 3.6. Economic Performance

During the production period, ReHS ≥ 60 was maintained for 62 days (56.4%) in AlimenWOW G2, compared with 0 days (0%) in Control 1 and 4 days (3.85%) in Control 2. Finishing rates were 90.6% for AlimenWOW G2, 90.8% for Control 1, and 87.4% for Control 2.

FCR values were 2.94, 2.99, and 3.19 in AlimenWOW G2, Control 1, and Control 2, respectively, indicating that FCR was 0.05 and 0.25 units higher in Control 1 and Control 2 than in AlimenWOW G2. ADG was about 70 g/day higher in Control 1 and Control 2 than in AlimenWOW G2; however, this difference was influenced by differences in slaughter-age distribution.

**Figure 6:**
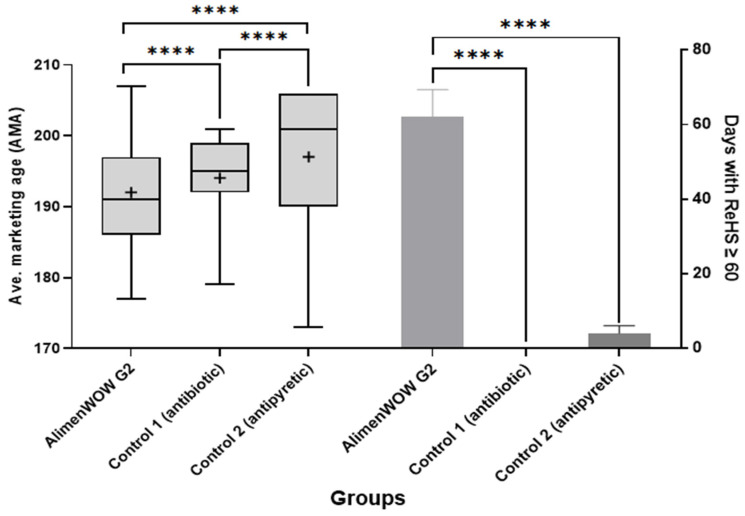
Comparison of average marketing age and days with ReHS ≥ 60 among experimental groups. The left panel presents a boxplot illustrating the distribution of average marketing age (AMA) for the AlimenWOW G2, Control 1, and Control 2 groups. The AlimenWOW G2 group exhibited the earliest median marketing age, whereas Control 1 and Control 2 showed progressively delayed distributions. The right panel compares the number of days during which Respiratory Health Status (ReHS) remained ≥60 throughout the rearing period. The AlimenWOW G2 group maintained ReHS ≥ 60 for a total of 62 days, while Control 1 and Control 2 maintained this threshold for only 0 and 4 days, respectively. Statistical analysis confirmed significant differences among the groups (*p* < 0.0001). In the AMA boxplot, the horizontal line indicates the median, the “+” marker denotes the mean, the box represents the interquartile range (IQR), and the whiskers indicate the minimum and maximum values. Asterisks indicate statistical significance (**** *p* < 0.0001).

Using the mean slaughter age of AlimenWOW G2 (191 days) as a reference, 238 pigs in AlimenWOW G2, 105 in Control 1, and 126 in Control 2 were slaughtered at or before 191 days. The numbers slaughtered after 191 days were 215, 349, and 311 in AlimenWOW G2, Control 1, and Control 2, respectively (Figure 6, *p* < 0.0001).

When feed costs during the 14-day interval from 177 to 191 days were compared, feed cost for AlimenWOW G2 was 44.12% and 52.95% of that for Control 1 and Control 2, respectively. At the batch level, total feed cost in AlimenWOW G2 was approximately 2.8% lower than in Control 1 and 5.3% lower than in Control 2.

## 4. Discussion

This field study comprehensively evaluated the impact of AlimenWOW, a rottlerin–lipid formulation, on respiratory health, mortality and wasting, PRRSV viral load, antibody responses, and economic outcomes in grower–finisher pigs under commercial conditions. Overall, AlimenWOW-treated groups (G1 and G2) showed improved respiratory indices, reduced viral loads, favorable antibody dynamics, and lower mortality and wasting rates compared with antibiotic- and antipyretic-treated controls, suggesting that AlimenWOW is a promising therapeutic adjunct to conventional management.

The AI-based SoundTalks^®^ system provided a robust framework for objective assessment of respiratory health. In AlimenWOW G1, which originated from a PRRSV-stable herd, ReHS remained ≥70 immediately after treatment and remained high throughout most of the observation period. In contrast, AlimenWOW G2, derived from a PRRSV-unstable farm and exhibiting low baseline ReHS (≤30), showed a marked improvement to 60–80 during weeks 3–4 following treatment, whereas Control 1 and Control 2, from the same unstable origin, remained at low ReHS (20–30) throughout. These findings indicate that AlimenWOW can both maintain favorable respiratory conditions in stable herds and facilitate recovery in high-risk groups with poor initial status.

The CCI, derived from ReHS, provided additional evidence of clinical benefit. From day 14 onward, AlimenWOW G2 maintained significantly lower CCI values than both control groups (*p* ≤ 0.0001), reflecting reduced cough frequency and milder clinical signs. The consistent pattern of high ReHS and low CCI in AlimenWOW-treated groups, even under identical barn conditions in Barn 1, suggests that pharmacological effects of AlimenWOW were more influential than environmental factors alone. In addition, other production batches housed in the same barn as AlimenWOW G2 showed similar respiratory conditions, and the barn was generally perceived by farm personnel as acoustically quiet, with few overt cough sounds.

Another limitation of this study is the absence of a vehicle-only (empty-liposome) control group. In this regard, the findings observed in the present study are generally consistent with prior reports showing that empty liposomes exhibit no antiviral activity or apparent non-specific effects [40].

In addition, as this was a field-based investigation conducted at the group level under commercial farm conditions, the lack of biological replication at the pen or barn level limits the generalizability of the findings.

Environmental conditions, particularly gas accumulation from slurry pits, are important co-determinants of respiratory disease in pigs [15,16,62]. Elevated ammonia and H_2_S levels can damage the respiratory mucosa, impair mucociliary clearance, and exacerbate PRRSV-associated lesions [17,20]. In our study, Control 1 and Control 2, housed in the same barn as AlimenWOW G1, showed persistently low ReHS, suggesting that PRRSV infection in combination with environmental gas stress contributed to poor respiratory outcomes. Despite this, AlimenWOW G1 and G2 maintained relatively favorable respiratory indices, implying that AlimenWOW conferred partial protection even under suboptimal environmental conditions.

The SoundTalks^®^-derived ReHS and CCI metrics also provided an objective basis for evaluating treatment efficacy. Ref. [63] reported that ReHS facilitates early detection of clinical signs, and Refs. [51,52,53] showed an association between ReHS and swIAV RNA detection. Ref. [54] demonstrated high accuracy and specificity for CNN–RNN–based cough detection systems in pigs. Taken together, these studies, along with our current findings, support the use of AI-based acoustic monitoring as a reliable tool for treatment evaluation under real-world conditions.

The patterns observed in antigen detection and antibody kinetics are consistent with the observed changes in ReHS and clinical signs. In AlimenWOW G1, PRRSV antigen remained at near-zero levels and ReHS was stable, indicating low viral circulation. In AlimenWOW G2, ReHS improved progressively over time, while viral titers declined and became undetectable by day 28. In contrast, control groups showed higher viral loads at day 14, consistent with persistent antigenic stimulation and relatively stable or sustained antibody titers.

PRRSV primarily targets porcine alveolar macrophages and uses CD163 and CD169 as key receptors [64,65]. Infection is characterized by early clinical and virological responses, but delayed adaptive immunity and potential persistence or subclinical carriage [66,67,68,69]. Hence, reductions in log_10_ TCID_50_/mL represent direct evidence of declining antigen burden. In our study, AlimenWOW G2 exhibited a stepwise decline in viral titers, culminating in complete negativity by day 28, whereas control groups maintained higher titers at day 14.

Previous experimental studies have suggested a potential antiviral framework for rottlerin-based formulations. Following oral administration, lipid-based formulations may improve the bioavailability of hydrophobic compounds such as rottlerin, and prior in vitro studies have proposed that rottlerin can interfere with actin-dependent processes involved in PRRSV entry into porcine macrophages. However, the present study did not directly evaluate pharmacokinetics, pulmonary distribution, cellular uptake, or intracellular target engagement in pigs, and, therefore, no mechanistic conclusions can be drawn from the current data.

Antibody kinetics further support the antiviral effect. In AlimenWOW G2, the S/P ratio peaked around day 14 and subsequently decreased by day 28, which may reflect an antibody response initially induced by antigen exposure that declined over time. In contrast, Control 1 and Control 2 maintained relatively stable S/P values, which may be associated with continued antigenic stimulation. [70] reported that changes in antibody titers during PRRSV infection can correlate with clinical improvement. In the present study, the decline in antibody titers observed in AlimenWOW-treated pigs occurred alongside improved clinical signs and reduced viral load.

Formulation characteristics are crucial for translational feasibility. Previous work using HSPC/cholesterol-based liposomes provided effective encapsulation of hydrophobic compounds but was limited by high cost and restricted production efficiency [40]. By contrast, the current AlimenWOW formulation uses soybean lecithin, an unsaturated phospholipid that is cost-effective and scalable, with high drug-loading capacity and physicochemical flexibility [71,72]. No significant differences in encapsulation efficiency have been reported between soybean and egg lecithin [72], and soybean lecithin–based mixtures retain stability and drug retention even in the absence of cholesterol. These features align well with the requirements of veterinary medicinal products, where low cost and high efficiency are essential.

The economic analysis highlights the practical relevance of AlimenWOW in PRRSV-unstable settings. Although ADG was somewhat higher in control groups, this difference stemmed partly from slaughter-age distribution, with a higher proportion of pigs in AlimenWOW G2 slaughtered earlier. More importantly, FCR was improved in AlimenWOW G2, resulting in lower feed costs. At the batch level, total feed costs in AlimenWOW G2 were approximately 2.8% lower than in Control 1 and 5.3% lower than in Control 2, indicating that the combined effects of improved FCR and earlier slaughter contributed to better overall economic performance, despite modest differences in ADG.

Finishing rates were similar across groups but generally lower than expected, suggesting a common factor affecting all pigs during the trial. Clinical observations and microbiological data pointed to edema disease associated with EDEC (stx2e/F18-positive *E. coli*) as an important contributor. EDEC can persist or reactivate during the grower–finisher stage [73,74,75]. Excess lysine in the initial diet likely activated lysine-dependent acid resistance mechanisms in pathogenic *E. coli*, enhancing survival in the stomach and facilitating intestinal colonization [76,77]. The rapid stabilization of mortality and clinical signs following diet correction strongly suggests that EDEC, rather than PRRSV, was the primary driver of reduced finishing rates in this cohort.

Beyond grower–finisher herds, AlimenWOW may also have potential in reproductive and vertical transmission control. PRRSV infection during mid-gestation is associated with severe vertical transmission and reproductive failure [5]. The timing overlaps with fetal testis descent, increasing the risk of viral establishment in fetal tissues and subsequent disease in suckling piglets. Administration of AlimenWOW around day 75 of gestation, alongside vaccination, may help prevent vertical transmission during high-risk periods. Likewise, early post-weaning piglets, which experience waning maternal antibodies [78,79], and pigs at transfer to finishing barns, where PRDC is common [80,81,82], represent additional windows in which strategic AlimenWOW use may mitigate PRRSV circulation and respiratory disease.

## 5. Conclusions

In summary, this field study demonstrates that AlimenWOW, a rottlerin–lipid formulation, exerts beneficial effects in PRRSV-affected grower–finisher herds, including improved AI-based respiratory health indices (ReHS, CCI), reduced mortality and wasting, decreased PRRSV viral load, and modulated antibody responses. These clinical and virological benefits translated into improved feed efficiency and lower feed costs under commercial conditions.

Strategic deployment of AlimenWOW, particularly during high-risk windows such as mid-gestation, early post-weaning, and transfer to finishing herds, has the potential to support farm-level PRRSV control by limiting vertical transmission and reducing viral circulation in nursery and grower–finisher pigs. As a complementary therapeutic approach to existing vaccination and antibiotic/antipyretic strategies, AlimenWOW represents a promising tool for mitigating PRRSV-associated economic losses and enhancing productivity in the swine industry. Further large-scale and long-term studies are warranted to confirm its efficacy across diverse production systems and PRRSV epidemiological contexts.

## Figures and Tables

**Table 1:** RT-qPCR conditions.

Step		Temperature (°C)	Time	Cycle
Reverse transcription		37	3 min	1
		50	20 s	1
Pre-denaturation		95	1 min	1
PCR thermocycling	Denaturation	95	5 s	40
	Annealing/Extension	56	30 s	

**Table 2:** Mortality and wasting after AlimenWOW administration.

Group	Mortality					Wasting				
	Week of Study				Total (%)	Week of Study				Total (%)
	1	2	3	4		1	2	3	4	
AlimenWOW G1	0	1	0	1	2 (0.4)	4	0	2	0	6 (1.2)
AlimenWOW G2	1	2	2	1	6 (1.2)	0	0	0	0	0 (0)
Control 1	2	3	5	3	13 (2.6)	2	2	5	0	9 (1.8)
Control 2	7	11	5	10	33 (6.6)	6	0	0	3	9 (1.8)

Groups were stratified according to ReHS criteria: AlimenWOW G1, ReHS ≥ 70; AlimenWOW G2 and control groups, ReHS ≤ 30.

## Data Availability

The data presented in this study are available on request from the corresponding author.

## Supplementary Materials

The following supporting information can be downloaded at: https://www.mdpi.com/article/10.3390/v18010072/s1, Supplementary Table S1: Distribution of pigs by severity scores of clinical signs in each group across study weeks.
