# Supplementary material for: AI-Based Respiratory Monitoring-Guided Evaluation of Rottlerin Therapy for PRRS in Grower–Finisher Pig Farms
Source: Viruses. 2026 Jan 4;18(1):72. doi: 10.3390/v18010072 (PMC12846505; doi:10.3390/v18010072)
Supplement: Supplementary file 1 [file viruses-18-00072-s001.zip › viruses-4059355-supplementary.pdf]

Supplementary Table S1. Distribution of pigs by severity scores of clinical signs in each group across study weeks.

Group			Score (n)				
			1	2	3	4	5
AlimenWOW G1	week of study	1	1	2	-	1	-
		2	-	-	-	-	1
		3	-	2	-	-	-
		4	-	-	-	-	1
AlimenWOW G2		1	-	-	-	-	1
		2	-	-	-	-	2
		3	-	-	-	-	2
		4	-	-	-	-	1
Control 1 (antibiotic)		1	1	-	1	-	2
		2	-	-	2	-	3
		3	-	1	2	2	5
		4	-	-	-	-	3
Control 2 (antipyretic)		1	1	-	4	1	7
		2	-	-	-	-	11
		3	-	-	-	-	5
		4	-	-	1	2	10

Clinical scores were assessed on a 1–5 scale, where scores 1–2 indicate mild signs and scores 3–5 indicate moderate-to-severe signs. Data are presented as number of pigs (percentage within each group per week).
